# Combined Stochastic Process and Value at Risk: A Real-World Information System Decision Case

**DOI:** 10.3390/e22010047

**Published:** 2019-12-30

**Authors:** Liang-Chuan Wu, Liang-Hong Wu, Fan-Yun Pai

**Affiliations:** 1 Institute of Technology Management, National Chung Hsing University, 250 Kuo Kuang Rd., Taichung 402, Taiwan; arthurwu@nchu.edu.tw; 2 Business School, University of Shanghai for Science and Technology, Shanghai 200093, China; 3 Department of Business Administration, National Changhua University of Education, Changhua 50007, Taiwan

**Keywords:** information system, stochastic process, uncertainty, value at risk (VaR)

## Abstract

In this study, we used a combined stochastic process and value-at-risk (VaR) method to examine an electronic commerce expansion decision. By modeling uncertain benefits as a stochastic process, maximum losses of alternative decisions were quantified and compared to help managers to make information system/information technology (IS/IT) project decisions. Our results, based on the maximum loss perspective, demonstrated that uncertainty plays a critical role in evaluating IS/IT projects. More importantly, the results illustrate that VaR serves as a useful tool in decision-making for managers to quantify the value of maximum possible loss and to help them reach decisions.

## 1. Introduction

Information technology (IT) is a course of action that collects, stores, processes, and transmits data [1]. The term “information technology” appeared in 1958 when Leavitt and Whisler [2] proclaimed that “new technology does not yet have a single established name”. Later, Mehrotra [3] referred to this term as IT. Numerous studies have attempted to link organizations’ investments in IT with overall competitive advantage in the pursuit of superior performance [4], and the results demonstrate that IT investments are one of the most critical managerial issues for organizations. Given the importance of IT investments, managers are keen to justify large IT expenses and to oversee one of the most risky decisions for organizations [5]. Value at risk (VaR), which originated from the insurance and banking field and stresses the maximum possible loss under uncertainty, has gained an increasing amount of attention in recent years from academics and practitioners such as the Basel Capital Accord for managing uncertainty in the banking industry. Despite its popularity in its original fields, the application of VaR in the information system (IS)/IT field is scarce. How to use VaR in IS/IT is still a new and untouched area.

In this study, we aimed to close the gap. We examined how to apply VaR for IS/IT investments and used real-world cases. In particular, we addressed the following questions:

(1) How can one design a formal evaluation framework for future studies and for practitioners to apply VaR in the IS/IT field?
(2) Does the concept of “minimizing benefits at risk” behind VaR provide different results than the concept of “maximizing fluctuating benefits” behind real options?

Leveraging the two fields can support academics and practitioners alike. For academics, IS/IT appraisal has always been important and including VaR in IS/IT can shed light on this topic. For practitioners, knowing how to use different perspectives to re-examine maximum loss under uncertainty can support top managers and help organizations improve appraisal decisions for the successful introduction of new technologies and, thus, gain competitiveness.

## 2. Literature Review

The IS/IT field has shown considerable interest in IT investment evaluation. Cost–benefit analysis is the main approach to justify difficult decisions [6,7,8,9,10,11,12,13]. However, traditional cost–benefit analysis such as the net present value (NPV) assumes static benefits and uses a pinpoint forecast over the project time, which is difficult for more complicated processes because they involve greater uncertainty [14,15].

However, researchers are increasingly discussing the importance of uncertainty for IS/IT investments. Real options consider uncertainty and have thus been applied to the IS/IT discipline. For example, Dos Santos [16] considered the value of future uncertainties to propose an option pricing model to determine the value of new technology investments. Benaroch and Kauffman [17] applied the Black–Scholes option pricing model to evaluate an e-bank investment. Kumar [18] demonstrated that the real options approach can be applied to IT investments using an Internet sales channel context. Real options can also evaluate Enterprise Resource Planning (ERP) investments. Taudes et al. [19] used a closed-form real options formula to examine the value of future opportunities for follow-on projects, such as e-commerce opportunities enabled by initial ERP investments. The estimated value based on real options evaluation is more realistic than the value based on conventional NPV estimates. Svavarsson [20] assessed the risk of IT investments and Wu et al. [21] used a real options approach to discuss uncertainties in ERP implementations. Lee et al. [22] found that real options are particularly valuable for information technology investments and can capture value that would be overlooked. In their view, rapidly advancing IT means that IT projects inherently contain high technical uncertainty as well as market uncertainty. In addition, high initial investments are often irreversible, making real options appropriate for IT investments. Park et al. [23] investigated real options thinking in a case regarding a decision to employ Radio Frequency Identification (RFID) technology, in which the technological uncertainty itself was considered the most critical issue in the investment decision. Benaroch [24] reported a real options decision-making process using the concept of real options for cybersecurity investment.

The real options theory stresses fluctuations of benefits, while VaR focuses on quantifying the maximum possible loss under uncertainty. VaR derives from the increasing regulatory demand for quantitative risk management tools due to the Asian financial market crash of 1987 and the disastrous losses from the derivatives trading of institutions, such as Lehman Brothers Holdings, Inc.; American International Group, Inc. (AIG); Merrill Lynch; and Long-Term Capital Management Funds. The VaR emerged as the most prominent measure of downside market risk [25,26] quantifying an upper bound on maximum losses that exceeds the VaR, which is the threshold with a target probability. Few IS/IT studies have discussed the significance of VaR for IS/IT evaluations. For example, Han et al. [27], as well as Benaroch et al. [28], described the decline of IS/IT investment value over a given period of time with a given probability as a result of uncertainty. Thus, defining the maximum possible loss can manage risks in a monetary way and control IS/IT projects at acceptable risk levels. Fielder et al. [29] discussed cybersecurity investment methodologies with the aim of maximizing the expected benefit from information security investment. In the formation of their research framework, the concept of VaR and maximum organizational loss was implicitly introduced.

Despite the comprehensive VaR concept, few studies have applied VaR in the IS/IT discipline because, unlike finance goods such as stock prices, IS/IT investment parameters cannot be directly obtained in public markets. Thus, simulation techniques must be employed to examine possible IS/IT benefits under uncertainty. Simulated benefits represent a financial time series. Therefore, the use of VaR inevitably involves additional mathematics tools, which can be complex for practitioners.

For the background, we developed an analytical uncertainty model based on well-proven finance mathematics. The model provides an easy yet insightful way for managers to understand IS/IT values. The next section elaborates the use of VaR and explains how the proposed model quantifies IS/IT processes under uncertainty.

## 3. The Electronic Commerce Decision

Here, we demonstrate a real-world case to illustrate our proposed method. The Yankee 24 shared electronic banking network of New England was making a decision regarding the deployment of electronic commerce services. Table 1 shows the raw data of the case [17]. The data included year and month, number of transactions, operational revenues, operational costs, and investment cost, which are the foundation for passive NPV analysis.

Facing uncertainty such as technology, organizational readiness, and competition, the timing of deploying the electronic commerce services was the main concern of the bank. Here, we demonstrate how future benefits under uncertainty can be derived based on the proposed method.

**Figure 1:**
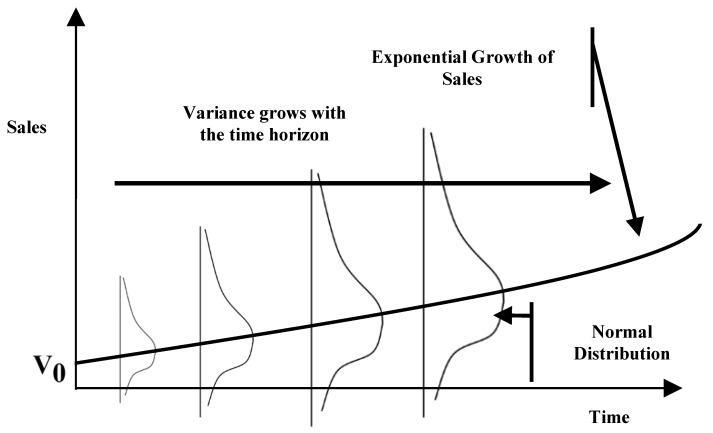
Uncertainties along exponential sales estimations of the electronic commerce decision.

**Figure 2:**
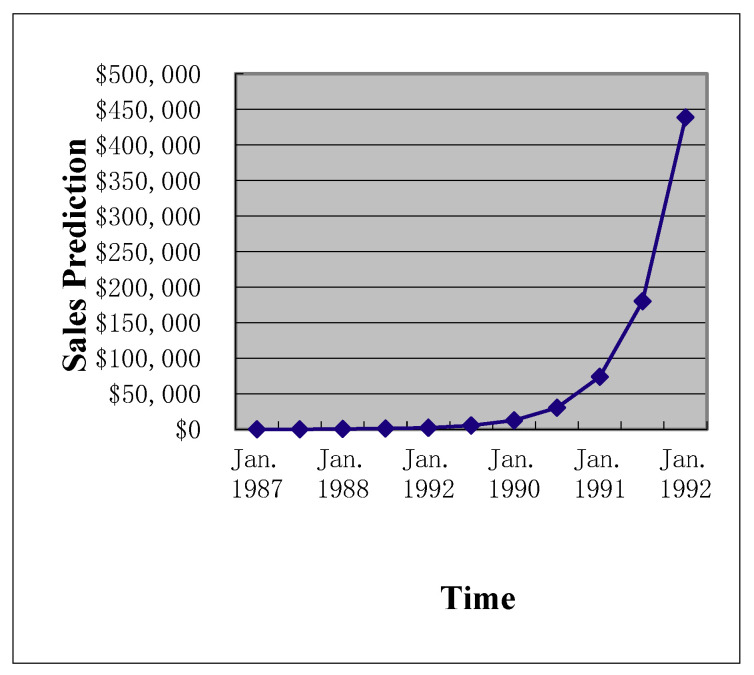
Prediction of future operational revenues.

**Figure 3:**
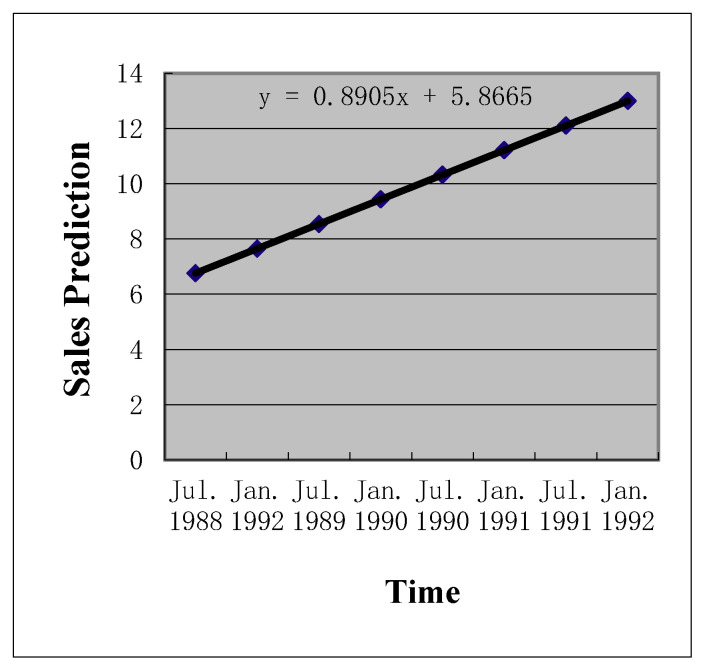
Nonstationary presentation after log-transformation.

In the first step, we modeled the uncertainty pattern reported in the bank expansion case. The value driver of the case, namely, the prediction of future operational revenues, demonstrated a clear exponential growth pattern (Figure 1 and Figure 2) and consisted of nonstationary data. Adopting the time series technique can log-transform the data into a nonstationary time series (Figure 3). We applied the time series approaches proposed by Tsay [30] to log-transform the exponential growth pattern to stationary data, since nonstationary data cannot be processed directly.

By applying the transform function, Formula (1) can deal with the nonstationary form of the prediction of future operational revenues after log-transformation (Table 2):

*F*(*X*) = *LN*(*X*). (1)

We followed the classical finance mathematics proposed by Dixit and Pindyck [31] to model the uncertainty. Under exponential growth of future operational revenues, geometric Brownian motion describes uncertainty after transformation to a stationary time series. We used geometric Brownian motion to model the uncertainty process, where uncertainty is divided into very small time slots (i.e., Δ*t*). In addition, the movement of the revenues was derived as:

(2) $$S=e^{x}\to X=\ln S,$$

where *S* is the value of the revenues, which increases exponentially over time. Therefore, the small movement in each small time slot Δ*t* is defined as follows:

(3) $$dX_{t}=udt+\sigma dW_{t},\;$$

where $dW_{t}=\varepsilon \sqrt{dt}$.

The geometric Brownian motion processes the property of:

(4) $$E(dX_{t})=(u-\sigma^{2}/2)dt,$$

(5) $$Var(X_{t})=\sigma^{2}dt,\;$$

where *u* is the drifting rate, and *s*^2^ is the variance.

Formula (3) indicates that the uncertain movement in each small time slot Δ*t* consists of two parts. The first one is the drift rate *u*, and the variance affects the second one. Solving this formula requires Ito’s lemma because it is a stochastic process. The closed-form solution derived from Formula (3) is:

(6) $$S_{t}=S_{0}{}^{e(u-\sigma^{2}/2)t+\sigma \sqrt{t}\varepsilon }\;\varepsilon \sim N(0,1).\;$$

Formula (6) is the analytical solution derived by modeling the uncertainty as the geometric Brownian motion and was used for the simulation of revenues under uncertainty in this electronic commerce decision case.

**Figure 4:**
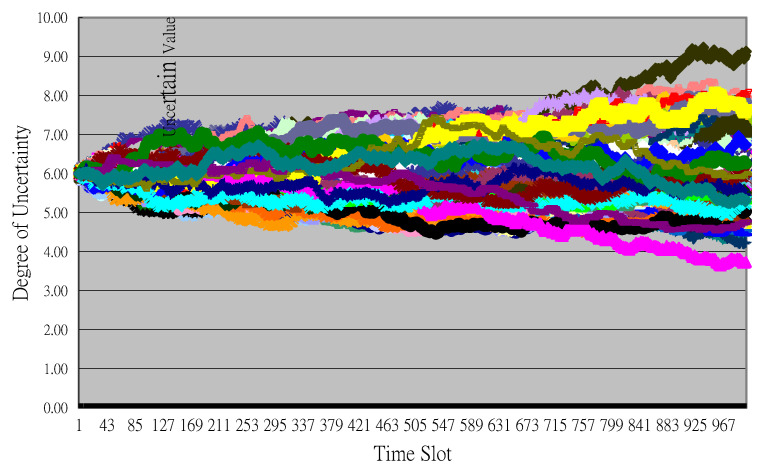
Partial Monte Carlo simulation results to decompose uncertainty.

Future uncertain benefits were simulated based on the model set in the previous step. The historical simulation method was used. We simulated future possible benefits using 10,000 Monte Carlo simulations for each period. Time complexity increased exponentially with the number of small time slots Δ*t*. The fewer the time slots Δ*t*, the quicker the simulation results were generated, but the standard deviation of the simulated results increased. Partial Monte Carlo simulation results for the July 1988 period demonstrated the pinpoint forecast used in traditional cost–benefit analysis with 1000 uncertainty paths to value uncertainty and VaR for benefits (Figure 4).

**Figure 5:**
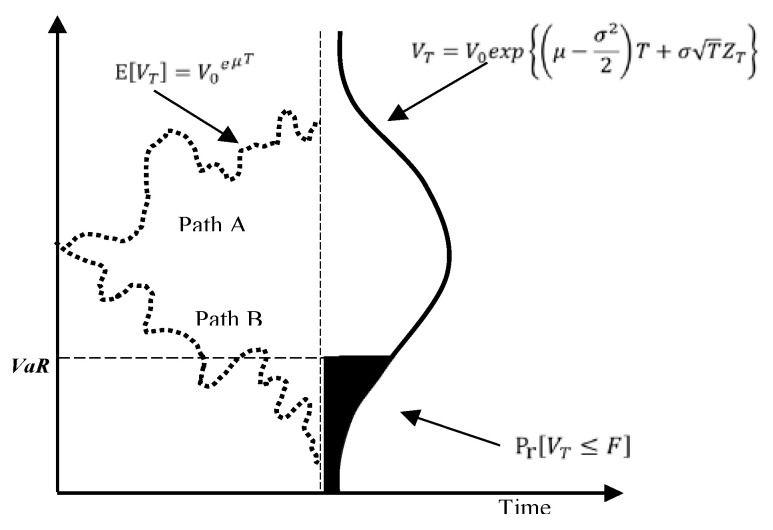
Value at risk in the proposed model.

The simulation techniques obtained value paths for the case values. With an initial value at time *t = 0*, the shadow varied over the whole project time (Figure 5). Different paths represent the possible value path subject to uncertainties that cannot be predicted at the initial evaluation stage. Monte Carlo simulations generated the possible paths. The shadow area in Figure 5 illustrates unfavorable possible paths of the case value. VaR is formally defined as follows: for a given time horizon *t* and confidence level *p*, the VaR of a project is the loss in value over the time horizon *t* that is exceeded with probability *1 − p* [32].

The possible tail of losses (i.e., the VaR level in Figure 5) represents the VaR value at a 95% confidence level.

## 4. Empirical Analysis

The confidence level was set and the VaR measure was computed. Comparing the VaR at each period can examine the profile of the electronic commerce decision with additional risk exposure information. Furthermore, optimal timing can be decided. The results from traditional pinpoint cost-benefit analysis and VaR analysis differed. In Table 3, the traditional cost–benefit analysis on the left-hand side has a pinpoint value. In column four of Table 3, the two numbers represent the maximum and minimum of the revenues. Even a sensitivity analysis considering 50% upper and lower fluctuations of the pinpoint value does not consider dynamic uncertainty. For example, the pinpoint value of benefits for July 1990 was fixed and was within $15,163–$45,489 without considering uncertainty. However, including uncertainty using geometric Brownian motion yielded max benefits during July 1990 of as much as $36,207 and, in the worst scenario, as little as $23,638 under a 60% confidence level.

To compare the effect of different confidence levels on the VaR analysis, five different confidence levels ranging from 60% to 99% were designed. The figures revealed that the greater the confidence level, the more critical the role of uncertainty became in bank expansion decisions. In column five of Table 3, the two numbers represent the maximum and minimum of the interval at a 60% confidence level. When the confidence level increased from 60% to 99%, the differences between these extremes increased. In an extreme scenario, maximum possible benefits can be up to 24 times higher than the pinpoint forecast (Table 4) and as little as 26% of management estimates under a 1–99% confidence level and 50% benefits uncertainty setting. Therefore, VaR reveals long-missing key information in IS/IT decision-making, such as the risk.

To facilitate management, the monetary term was used to demonstrate the cost and importance of risk. Table 4 summarizes bank expansion with regard to maximum possible loss under a 1 − α% confidence level. For the 60% confidence level, an extreme loss can be as high as $172,465, much greater than the $76,766 the management estimated. Under 1–99% confidence, extreme but still possible, VaR reported huge loss information (Table 4), which stresses the importance of extreme cases for organizations to improve risk management.

**Figure 6:**
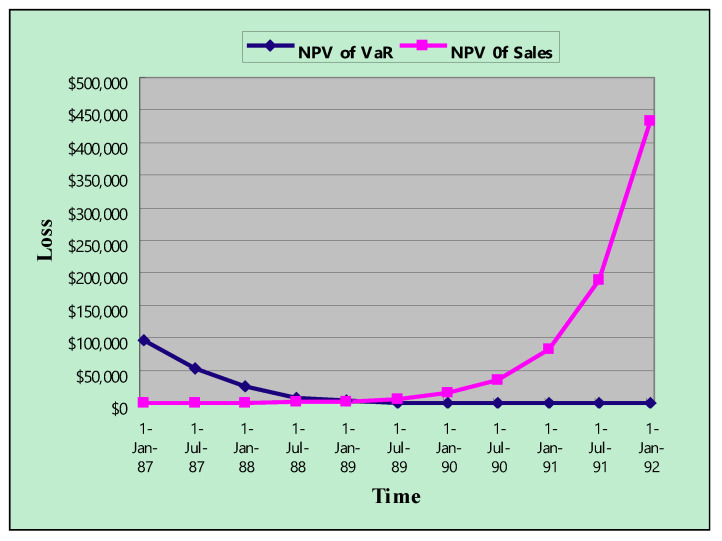
The opportunity cost and value at risk (VaR) over the project life (Years are abbreviated. For example, 87 represents 1987).

To examine the benefits of uncertainty for banking expansion decisions, three different variance levels for benefits (i.e., 50%, 75%, and 100%) were used (Table A1 in Appendix A and Table A2 in Appendix B). Furthermore, it was of interest to examine how VaR can be used to assess the optimal decision period of the bank expansion decision. VaR derives from future uncertainty and becomes smaller with clearer information, that is, the later the deployment decision, the smaller the VaR. However, from an investment opportunity perspective, first-mover advantage for market shares is smaller when the longer investment decisions are delayed. Table 5 demonstrates and Figure 6 depicts the opportunity cost and VaR over the project life (60% confidence level and 50% benefit uncertainty).

**Figure 7:**
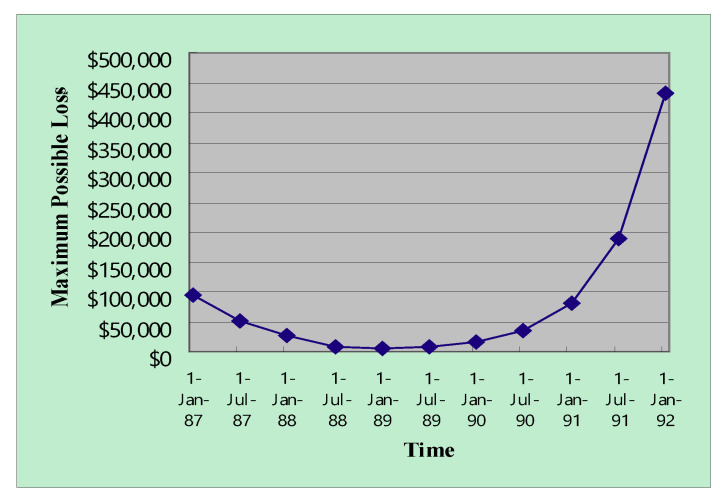
The combined maximum value of loss over the project life (60% confidence level and 50% benefit uncertainty) (Years are abbreviated. For example, 87 represents 1987).

**Figure 8:**
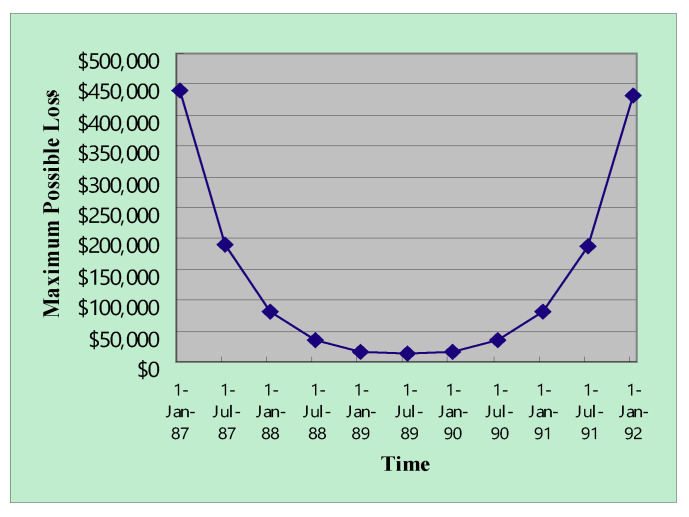
The combined maximum value of loss over the project life (99% confidence level and 100% benefit uncertainty) (Years are abbreviated. For example, 87 represents 1987).

Adding the two functions resulted in a tradeoff (Figure 7 and Figure 8). Two tradeoff facts explain the U-shaped loss function. An early deployment of the e-commerce service can lead to first-mover advantage but bears huge uncertainty. The combined maximum value of the loss function over the project life illustrates the optimal timing for deploying e-commerce technology in terms of possible loss (Figure 7 and Figure 8).

To avoid risk and possible harmful outcomes from uncertainty, Table 6 lists optimal timing. With a 60% confidence level and 75% benefit variance, the optimal timing for the POS debit service decision was January 1989. When the confidence level was 99%, July 1989 was the optimal timing for the decision.

The benefit uncertainty and the confidence level of the VaR both influenced optimal timing (Figure 7 and Figure 8). Increasing the confidence level of VaR increased the left side of the U shape and shifted the optimal timing to the right. Too early entries into markets would bear too much uncertainty because uncertain demands resolve over time, and potential failures can represent costs to Yankee 24. However, Yankee 24 may not enjoy market shares when entering too late. Our analysis suggests that July 1989 was the optimal timing for the e-commerce service for the Yankee 24 shared electronic banking network of New England, which is very similar to results from the literature [17], which suggested January 1990. Figure 6 demonstrates the similarity of the two results. Even though different approaches were used, our results were validated by the timing suggestion reported in the literature because the bottom of the U-shaped function fell approximately on July 1989 and January 1990.

## 5. Conclusions and Implications

In the present study, we employed the VaR in a real-world case of the Yankee 24 bank e-commerce service expansion decision. A normative framework illustrated how VaR can be applied to different disciplines. Using interdisciplinary tools such as geometric Brownian motion modeling, time series analysis, and Monte Carlo simulations demonstrated that VaR is a promising developing tool in the insurance and banking industry and can shed light on the IS/IT field.

This study contributes to academic research in three ways. First, the proposed framework is original and provides insights that contribute to the IS/IT literature. Second, the use of VaR can support IS/IT evaluation, which is important for appraising expenses. Third, a real-world case was revisited based on avoiding maximum loss. The concept of seeking “maximum benefits” has long been used in the IS/IT discipline, and our results have been validated.

The study is also interesting for practitioners because a normative framework was used for a real-world case and can be directly applied to projects. The results also stress risk as a cost and support practitioners in re-examining the role of risk inherent to IS/IT projects. Furthermore, evaluating IS/IT projects is increasingly important for managers and this study provides a reference case.

This study used a geometric Brownian motion model, and how this model is robust to the violation of its assumptions can be further studied. One possible extension of this work is to use alternative processes such as extreme value regression models [33] that can be applied when the assumptions are violated. It would also be interesting to study how to combine information from different sources [34,35]. Moreover, the back-testing method [35] is interesting for future studies. The in-sample calculated VaR could be compared with the out-of-sample values to generate a predictive accuracy measure. One limitation of this study is that, unlike time series data for financial assets, data for IT investments are limited and evaluating the goodness of the risk estimation still needs to be further explored by researchers. Several approaches can improve this study. First, because the quality of the VaR depends on how the uncertainty model fits the case property, future studies can use different models to describe uncertainty that map different IS/IT project properties. Second, further research could generate longitudinal data for evaluation and exploration of the results.

## Figures and Tables

**Table 1:** Passive net present value (NPV) analysis of the electronic commerce decision.

Month	Year	Number of Transactions	Operational Revenues	Operational Costs	Net Revenues	Investment Costs	Cash Flows
January	1987	0	$0	$0	$0	$400,000	−$400,000
July	1987	0	$0	$0	$0	$0	$0
January	1988	3532	$353	$20,000	−$19,647	$0	−$19,647
July	1988	8606	$861	$20,000	−$19,139	$0	−$19,139
January	1989	20,969	$2097	$20,000	−$17,903	$0	−$17,903
July	1989	51,088	$5109	$20,000	−$14,891	$0	−$14,891
January	1990	124,470	$12,447	$20,000	−$7553	$0	−$7553
July	1990	303,258	$30,326	$20,000	$10,326	$0	$10,326
January	1991	738,857	$73,886	$20,000	$53,886	$0	$53,886
July	1991	1,800,149	$180,015	$20,000	$160,015	$0	$160,015
January	1992	4,385,877	$438,588	$20,000	$418,588	$0	$418,588

**Table 2:** The transformation from nonstationary to stationary data.

	Month	Year	Number of Transactions	Operational Revenues (*X*)	Formula (1) Transformed by *X* = ln(*x*)	Value Return by *X* = e
0	January	1987	0	$0	0	$0
1	July	1987	0	$0	0	$0
2	January	1988	3532	$353	5.8	$353
3	July	1988	8606	$861	6.8	$860
4	January	1989	20,969	$2097	7.6	$2095
5	July	1989	51,088	$5109	8.5	$5105
6	January	1990	124,470	$12,447	9.4	$12,438
7	July	1990	303,258	$30,326	10.3	$30,305
8	January	1991	738,857	$73,886	11.2	$73,834
9	July	1991	1,800,149	$180,015	12.1	$179,890
10	January	1992	4,385,877	$438,588	13.0	$438,283

**Table 3:** The value-at-risk analysis (the bold numbers represent the minimum of the revenues).

	Source of Uncertainty Pinpoint Prediction for Sales		Sensitivity Analysis (50% Fluctuations)	Value at Risk Sales Uncertainty Modeled with Geometric Brownian Motion (Variance = 50%)				
	Sales Prediction	Operational Revenues	Pinpoint Max/Min Value	Upper/Lower Bound of Continuous Value				
				60%	80%	90%	95%	99%
**January 1987**	0	$0	$0	$0	$0	$0	$0	$0
**July 1987**	0	$0	$0	$0	$0	$0	$0	$0
**January 1988**	3532	$353	$353	$353	$353	$353	$353	$353
**July 1988**	8606	$861	$1292	$1046	$1323	$1543	$1828	$2239
			$431	**$855**	**$685**	**$545**	**$477**	**$393**
**January 1989**	20,969	$2097	$3147	$2362	$3221	$4184	$4804	$7377
			$1049	**$1781**	**$1346**	**$1112**	**$942**	**$798**
**July 1989**	51,088	$5109	$7664	$5857	$8860	$13,093	$16,133	$25,615
			$2555	**$4449**	**$2843**	**$2305**	**$1891**	**$1223**
**January 1990**	124,470	$12,447	$18,671	$13,386	$25,867	$39,602	$54,920	$97,905
			$6224	**$9446**	**$6595**	**$4672**	**$3732**	**$2542**
**July 1990**	303,258	$30,326	$45,489	$36,207	$57,569	$90,295	$126,856	$226,619
			$15,163	**$23,638**	**$15,743**	**$10,946**	**$9034**	**$5836**
**January 1991**	738,857	$73,886	$110,829	$75,116	$137,576	$193,056	$287,976	$658,149
			$36,943	**$46,319**	**$31,977**	**$22,249**	**$15,476**	**$11,090**
**July 1991**	1,800,149	$180,015	$270,023	$236,519	$473,823	$832,029	$1,209,168	$2,294,620
			$90,008	**$137,739**	**$78,261**	**$52,939**	**$43,052**	**$28,289**
**January 1992**	4,385,877	$438,588	$657,882	$606,452	$1,179,795	$2,000,229	$3,103,931	$15,808,640
			$219,294	**$365,787**	**$235,577**	**$168,381**	**$95,609**	**$58,988**

**Table 4:** Present value of maximum possible loss under 1 − α% confidence level.

	Pinpoint Prediction for Sales	Investment Cost	Operational Costs	Value at Risk Sales Uncertainty Modeled with Geometric Brownian Motion (Variance = 50%)					
				60%		80%	90%	95%	99%
				Sales	Net Cash Flows				
**January 1987**	**$0**	**−$400,000**	**-**	**$0**	**−$40,000**	**$0**	$0	$0	$0
**July 1987**	$0	-	-	$0	$0	$0	$0	$0	$0
**January 1988**	$353	-	−$20,000	$353	−$19,647	$353	$353	$353	$353
**July 1988**	$861	-	−$20,000	$855	−$19,145	$685	$545	$477	$393
**January 1989**	$2097	-	−$20,000	$1781	−$18,219	$1346	$1112	$942	$798
**July 1989**	$5109	-	−$20,000	$4449	−$15,551	$2843	$2305	$1891	$1223
**January 1990**	$12,447	-	−$20,000	$9446	−$10,554	$6595	$4672	$3732	$2542
**July 1990**	$30,326	-	−$20,000	$23,638	$3638	$15,743	$10,946	$9034	$5836
**January 1991**	$73,886	-	−$20,000	$46,319	$26,319	$31,977	$22,249	$15,476	$11,090
**July 1991**	$180,015	-	−$20,000	$137,739	$117,739	$78,261	$52,939	$43,052	$28,289
**January 1992**	$438,588	-	−$20,000	$365,787	$345,787	$235,577	$168,381	$95,609	$58,988
**NPV** **(Pinpoint Estimation))**	−$76,766	**NPV** **(in Terms of Maximum Possible Loss, under 1−α% Confidence)**			−$172,465	−$305,877	−$373,573	−$429,936	−$467,659

**Table 5:** Tradeoff of the Point of Sales (POS) debit service deployment decision (60% confident level and 50% benefit uncertainty).

Periods Deferrable	Date	Opportunity Cost (NPV of Sales)	VaR	Total Possible Maximum Loss
0	January 1987	$95,700	$0	$95,700
0.5	July 1987	$52,609	$0	$52,609
1	January 1988	$26,084	$314	$26,399
1.5	July 1988	$7751	$1037	$8788
2	January 1989	$3036	$2698	$5734
2.5	July 1989	$793	$6516	$7309
3	January 1990	$271	$15,290	$15,561
3.5	July 1990	$5	$35,459	$35,464
4	January 1991	$0	$81,816	$81,816
4.5	July 1991	$0	$188,367	$188,367
5	January 1992	$0	$433,272	$433,272

**Table 6:** The decision matrix for the deferral period.

	Benefit Variance 50%	Benefit Variance 75%	Benefit Variance 100%
VaR (Confidence Level: 60%)	January 1989	January 1989	January 1989
VaR (Confidence Level: 99%)	↑ July 1989 ↓	↑ July 1989 ↓	↑ July 1989 ↓

## Appendix A. Supplementary Data (Variance = 75%)

**Table A1:** The value-at-risk analysis (variance = 75%).

	Pinpoint Prediction for Sales	Investment Cost	Operational Costs	Value at Risk Sales Uncertainty Modeled with Geometric Brownian Motion (Variance = 75%)				
				60%	80%	90%	95%	99%
**January 1987**	**$0**	**−$400,000**	-	$0	$0	$0	$0	$0
**July 1987**	$0	-	-	$0	$0	$0	$0	$0
**January 1988**	$353	-	−$20,000	$353	$353	$353	$353	$353
**July 1988**	$861	-	−$20,000	$771	$561	$455	$381	$262
**January 1989**	$2097	-	−$20,000	$1812	$1068	$807	$698	$444
**July 1989**	$5109	-	−$20,000	$3524	$2100	$1386	$1119	$643
**January 1990**	$12,447	-	−$20,000	$8518	$5073	$3571	$2773	$1847
**July 1990**	$30,326	-	−$20,000	$20,963	$10,958	$7699	$5314	$3347
**January 1991**	$73,886	-	−$20,000	$46,811	$22,175	$15,785	$11,624	$7193
**July 1991**	$180,015	-	−$20,000	$120,899	$51,472	$32,159	$23,189	$13,769
**January 1992**	$438,588	-	−$20,000	$329,482	$146,501	$86,522	$56,188	$33,836

## Appendix B. Supplementary Data (Variance = 100%)

**Table A2:** The value-at-risk analysis (variance = 100%).

	Pinpoint Prediction for Sales	Investment Cost	Operational Costs	Value at Risk Sales Uncertainty Modeled with Geometric Brownian Motion (Variance = 100%)				
				60%	80%	90%	95%	99%
**January 1987**	$0	−$400,000	-	$0	$0	$0	$0	$0
**July 1987**	$0	-	-	$0	$0	$0	$0	$0
**January 1988**	$353	-	−$20,000	$353	$353	$353	$353	$353
**July 1988**	$861	-	−$20,000	$721	$452	$347	$288	$165
**January 1989**	$2097	-	−$20,000	$1661	$1029	$722	$606	$330
**July 1989**	$5109	-	−$20,000	$3181	$1608	$1065	$723	$409
**January 1990**	$12,447	-	−$20,000	$10,073	$4317	$2281	$1427	$777
**July 1990**	$30,326	-	−$20,000	$18,137	$9420	$5513	$3924	$1847
**January 1991**	$73,886	-	−$20,000	$33,807	$14,523	$8583	$5563	$3058
**July 1991**	$180,015	-	−$20,000	$122,061	$42,459	$22,216	$15,233	$8048
**January 1992**	$438,588	-	−1$20,000	$243,754	$93,322	$50,802	$32,387	$17,497
